# Peripheral facial nerve palsy in children: clinical manifestations, treatment and prognosis

**DOI:** 10.1186/s41983-022-00596-1

**Published:** 2022-12-09

**Authors:** Serap Bilge, Gülen Gül Mert, M. Özlem Hergüner, Faruk İncecik, Özgür Sürmelioğlu, Sevcan Bilen, Levent Yılmaz

**Affiliations:** 1 Department of Pediatric Neurology, Faculty of Medicine, Çukurova University, Adana, Turkey; 2 Department of Ear, Nose &Throat, Faculty of Medicine, Çukurova University, Adana, Turkey; 3 Department of Pediatric Emergency, Faculty of Medicine, Çukurova University, Adana, Turkey

**Keywords:** Facial nerve paralysis, Clinical findings, Treatment, Prognosis

## Abstract

**Background:**

Sudden onset of unilateral weakness of the upper and lower muscles of one side of the face is defined as peripheral facial nerve palsy. Peripheral facial nerve palsy is often idiopathic and sometimes it could be due to infectious, traumatic, neoplastic, and immune causes. This study aimed to report the clinical manifestation, evaluation, and prognosis in children with peripheral facial nerve palsy.

**Methods:**

57 children under 18 years of age diagnosed with peripheral facial nerve palsy at Çukurova University, Balcalı Hospital, between January 2018 and September 2021, were included in the study.

**Results:**

The mean age of the children at the time of diagnosis was 9.6 ± 7, 4 years. Thirty-two (56.1%) of the patients were female and 25 (43.9%) were male. A total of 57 patients were diagnosed with peripheral facial nerve palsy and categorized into many groups by etiology: idiopathic Bell’s palsy in 27 (47.5%), infectious in 11 (19.2%), traumatic in 6 (10.5%), and others (due to congenital, immune, neoplastic, Melkersson–Rosenthal syndrome, drug toxicity, and iatrogenic causes) in 13 (22.8%). Forty-six of the children achieved full recovery under oral steroids within 1–7 months. Four patients with acute leukemia, myelodysplastic syndrome, Mobius syndrome and trauma did not recover and two patients (schwannoma, trauma) showed partial improvement. Five patients could not come to follow-up control.

**Conclusion:**

Peripheral facial nerve palsy is a rare condition in children with different causes. It could be idiopathic, congenital, or due to infectious, traumatic, neoplastic, and immune reasons. So, when a child presents with facial palsy, a complete clinical history and a detailed clinical examination are recommended. Giving attention to the red flag is very important. Peripheral facial nerve palsy in children is considered to have a good prognosis.

## Background

The sudden onset of unilateral weakness of the upper and lower muscles of one side of the face is defined as peripheral facial nerve palsy (PFNP). This condition is often idiopathic (formerly called Bell palsy) and primarily unilateral. This condition is first described by a Scottish surgeon named Charles Bell in 1821. An estimated 15–40 per 100,000 people per year are affected [1–4].

The pathophysiology of PFNP remains unclear. Compression of the facial nerve through the facial canal, especially the narrow labyrinthine segment, is the most acceptable theory. Any inflammatory, demyelinating, and ischemic or compressive process in this area may impair neural conduction [5–8]. Viral infection is the most commonly accepted theory behind PFNP, and the herpes simplex virus is the most frequently implicated [8–11]. Other suggested theories include autoimmunity, inflammation, and microvascular disease such as diabetes mellitus [8, 12–20]. The degree of deficit in PFNP is classified into six grades using the House–Brackmann scale (HBS) (Table 1). In this paper, we aimed to report the clinical manifestations, evaluation, treatment, and prognosis of children with PFNP admitted to Çukurova University, Balcalı Hospital.

**Table 1:** House–Brackmann facial paralysis scale [1]

Grade	Impairment
I	Normal
II	Mild dysfunction (slight weakness, normal symmetry at rest)
III	Moderate dysfunction (obvious but not disfiguring weakness with synkinesis, complete eye closure with maximal effort, good forehead movement
IV	Moderately severe dysfunction (obvious and disfiguring asymmetry, significant synkinesis) incomplete eye closure, moderate forehead movement
V	Severe dysfunction (barely perceptible motion)
VI	Total paralysis (no movement)

## Materials and methods

**Fig. 1:**
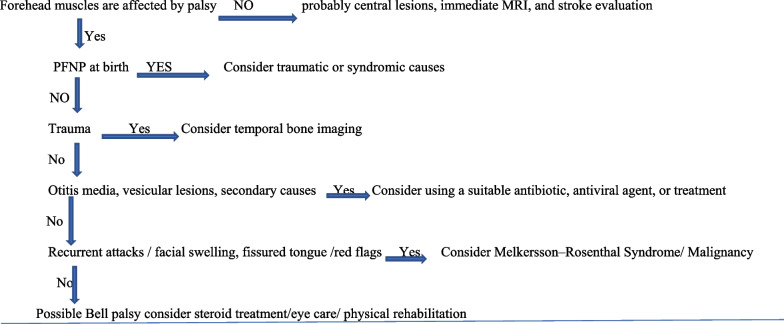
Algorithm for aid in diagnosing children with facial nerve palsy

The medical records of children under 18 years of age who approached Çukurova University-Balcalı Training and Research Hospital from January 2018 to September 2021 were retrospectively reviewed to detect the following ICD-10 codes: G51, facial nerve disorders; G51.0, Bell’s palsy; G51.8, other disorders of the facial nerve; G51.9, disorders of facial nerve, unspecified; facial nerve dysfunction undiagnosed. The inclusion criteria were acute isolated unilateral peripheral or bilateral peripheral facial nerve palsy and a follow-up period until recovery or at least 9 months. All patients were evaluated within 72 h of the onset of paralysis and also examined by an ophthalmologist, pediatric neurologist, and otolaryngologist during the initial evaluation. Medical records were reviewed to determine the age, gender, side of involvement, family history, etiology, results of diagnostic investigations, treatment modalities, and outcomes. The grade of facial nerve dysfunction was evaluated according to the House–Brackmann Facial Nerve Grading Scale (HBS) (Table 1). The Hospital Ethical Committee approved the study. Formal signed consent was taken from all parents or guardians. Complete blood count, viral and bacterial serology, B12, folate, parathyroid, thyroid hormone levels, C-reactive protein, blood sedimentation rate, and serum glucose levels were recorded. The diagnosis of Bell’s palsy was made when other causes of peripheral facial palsy such as viral infection, otitis media, trauma, Ramsay Hunt syndrome, or any secondary causes were excluded by history, physical examination, and diagnostic investigations. Children with atypical signs and symptoms of systemic or additional neurological involvement were further investigated. Diagnostic visualization of the brain and internal acoustic canal was performed by magnetic resonance imaging (MRI-1.5T MR Philip Intra 2008 model)) and computed tomography (CT-Siemens Somatom 16 Ct) when clinically indicated as in cases of trauma were done [6, 21]. Children with Bell’s palsy were treated for 7–10 days with oral prednisolone (1–2 mg/kg/day, max dose 60 mg/day); prednisolone treatment was subsequently tapered off within the next 3–5 days. All the treatment protocols were started in the first week of the initial symptoms. When a viral infection is suspected, antiviral medication was initiated. Artificial tears and facial exercises were also suggested by the ophthalmologist and physiotherapist. All patients were re-examined by the same pediatric neurologist on days 7 and 15 for any evolving problem and at the 1st, 2nd, and 6th months with respect to clinical outcome. Electroneuromyography was performed in all unrecovered, severe, and atypical cases (22 Nihon Kohden EMG (Neuropack MEB 9600) machine was used. Nasopharyngeal swab reverse transcriptase-polymerase chain reaction (PCR) was performed for SARS-CoV-2 in patients with fever or myalgia and for the patients who approached during the pandemic period (Fig. 1).

## Results

Fifty-seven children under 18 years of age, diagnosed with peripheral facial nerve palsy at Çukurova University, Balcalı Hospital between January 2018 and September 2021, were included in the study. The mean age of the children at the time of diagnosis was 9.6 ± 7,4 years. Thirty-two (56.1%) of the patients were female, and 25 (43.9%) were male. All the patients had unilateral paralysis except one, 27 (48.3%) on the right side and 29 (51.75%) on the left side of the face. Only 1 patient diagnosed with Guillain–Barre syndrome had bilateral palsy In general, the HBS grade ranged from 2 to 6; one child presented with HBS grade 6, another one with grade 5, 28 (49.1%) with grade 4, 21 (36.8%) with grade 3, and 2 (3.5%) with grade 2. The physical and neurological examinations other than facial nerve palsy were normal in all children except eight patients; they had neurological deficits and anomalies such as acute weakness of the extremities, change in consciousness, herpetic lesions, and proptosis. Additional hearing loss was reported in 4 patients (5.9%).

A total of 57 patients were diagnosed with PFNP and categorized into many groups: idiopathic Bell’s palsy in 27 (47.5%), infectious( CMV, EBV, varicella-zoster, mycoplasma pneumonia, otitis media, otitis externa, and abscess led to PFNP) in 11 (19.2%), a trauma in 6 (10.5%), and other reasons (congenital, immune, neoplastic (leukemia, myelodysplastic syndromes, solid mass), Melkersson–Rosenthal syndrome, tacrolimus toxicity, and iatrogenic causes) in 13(22.8%). Infections before PFNP were present in some patients, otitis media in 7, upper respiratory infection in 7, and acute gastroenteritis in 2 patients.

All the patients had a single attack of paralysis, except two patients had two attacks (Melkersson–Rosenthal syndrome and idiopathic case with low B12). The level of B12 was under 200 pg/mL in 14 patients and 25 patients were > 200 pg/mL. Nine patients had abnormal MRI findings, while 36 revealed normal cerebral and auricular MRI. Four of the 9 patients had enhancement of facial nerve, a patient had a schwannoma, and a stylomastoid mass was seen in another one, a transverse sinus thrombosis, otomastoiditis, and absence of facial nerve nucleus were reported in other patients. The computed tomography showed a fracture of the temporal bone in two patients and sinusitis in one, and effusion of the middle ear in another one. The serological tests showed abnormal results in 5 patients, such as CMV in 2 patients and EBV, varicella-zoster, and mycoplasma pneumonia in each patient. No SARS-CoV-2 could be detected in any patient with PFNP.

Forty-six of the children achieved full recovery under oral steroids or other required drugs according to the etiology within 1–7 months post-treatment, while 2 patients diagnosed with trauma and schwannoma expressed partial improvement. The patient with schwannoma partially recovered from HBS grade 5 to grade 3. The other four patients (acute leukemia, myelodysplastic syndrome, Mobius syndrome, traumatic birth) did not recover even after one year of follow-up, and the first two died within a few months. Five patients with regular improvement till 9 months could not come to follow-up control, so the results were unknown in these patients wether complete recovery or partial improvement was seen since recovery can been seen cases with PFNP even after one year.

Three children underwent surgery because of schwannoma in one patient and temporal bone trauma in 2 patients. A girl who presented with ipsilateral lip swelling and PFNP was diagnosed with Melkersson–Rosenthal syndrome. However, the treatment for this syndrome is still controversial, her facial function recovered completely under therapy with methylprednisolone.

Electrophysiological analyses such as electroneurography (ENoG) and needle EMG (nEMG) could be done in only 12 patients. Denervation of the facial nerve was seen in these patients, but no detailed reports could be seen in their medical records.

## Discussion

A complete clinical history and a detailed clinical, otoscopic examination, audiogram tests, and complete blood count are mandatory in all children presenting with PFNP. In the absence of additional symptoms or specific findings on physical examination, the utility of further investigation is debatable. Manning et al. studied the causes of PFNP in 61 children and concluded that 50% of cases had Bell’s palsy, 14.8% had infections, 11.5% had injuries, and 3.3% had congenital problems. Grundfos et al. concluded that 84% of children had PFNP due to a specific etiology, and only 16% had Bell’s palsy as a diagnosis of exclusion. Particular causes of facial nerve palsy included injuries (24%), otitis media (16%), infections (12%), neoplasias (12%), and congenital anomalies (8%). In our study, no cause could be detected in 47.3% of the patients, infection was seen in 11(19.2%), a trauma in 6(10.5%), and others in 13(22.8%) were due to congenital, immune, neoplastic, Melkersson–Rosenthal syndrome, drugs toxicity and iatrogenic causes which were similar with Manning et al. results [23, 24].

A 10-year-old girl presented to our emergency department with facial immobility and an inability to close her eyes or move part of her mouth. Her symptoms started one day previously with severe pain in the shoulder and extremities. Physical examination revealed PFNP and mild bilateral weakness of the extremities, hypoesthesia, remarkable deep-sensation deficit in lower and upper extremities, and loss of Achilles reflex. Diagnosis of Guillain–Barre syndrome was established based on physical examination and electroneurophysiological findings. After the treatment, PFNP recovered within 3 months. There are also some reports in the literature regarding patients with GBS presenting only with PFNP without any other symptoms. Hence detailed examination is critical in every case of FNP [25].

Unilateral facial nerve palsy can be a rare presenting symptom of leukemia or leukemic relapse. In our study 1, patients were diagnosed with AML after developing PFNP. In his examination, paleness and proptosis and abnormal CBC findings were red flags. Typical MRI findings cannot exclude the diagnosis of leukemia or leukemic relapse. Developing a focal neurological deficit in a patient with known leukemia warrants rigorous investigation and close surveillance for possible central nervous system relapse. In neoplastic facial palsy, the prognosis depends on the type and stage of neoplasm, and the therapeutic protocol applied in each case. PFNP in our patient recovered within four months, and the patient had bone marrow transplantation [8, 26, 27].

Inappropriate treatment of acute otitis media can cause facial nerve palsy, mastoiditis, labyrinthitis. Ramsay Hunt syndrome is caused by the varicella-zoster virus (VZV) reactivation, which lies in the sensory ganglion after primary infection. The syndrome is characterized by facial nerve palsy associated with a painful vesicular eruption within the external auditory canal and vestibulocochlear dysfunction (sensorineural hearing loss, vertigo, nystagmus, and ataxia). EBV, CMV, and mycoplasma pneumonia are also among the infectious causes [6, 11, 14]. In our study, CMV, EBV, varicella-zoster, mycoplasma pneumonia, otitis media, otitis externa, and abscess led to PFNP in 11(19.2%) patients. Patients with PFNP caused by infections recovered within 0.5–3 months. Some cases such as Ramsey Hunt syndrome may have a worse prognosis. All these patients received a combination treatment of corticosteroid and acyclovir in herpes or VZV infection, corticosteroid and ceftriaxone in complicated otitis media, and gancyclovir in CMV. PFNP, due to infectious causes, recovered within three months with the support of 2 weeks of corticosteroid and physical rehabilitation till recovery. The ongoing COVID-19 pandemic has affected millions of people worldwide and revealed several neurological syndromes related to this infection. But no cases of PFNP due to SARS-CoV-2 could be detected in our center.

Generally, the prognosis of facial nerve palsy depends on the cause. When caused by perinatal injury, congenital facial nerve palsy has an excellent prognosis without treatment, while it is permanent when caused by congenital dysplastic structural reasons. In this case, paralysis can be partly improved with plastic surgery procedures. Patients with traumatic facial nerve palsy recover within 30 months, with better results when the paralysis is partial or treated with steroids, physiotherapy, or surgical procedures [4]. In our study, the patient who had PFNP due to trauma recovered within seven months due to a mild injury, immediate surgical intervention, and extensive support of physical rehabilitation therapy were supplied. But PFNP in the patient with birth trauma didn't show any improvement. This situation could be due to the severe degree of birth trauma.

One of the rare reasons for FNP in children is hypertension. The ignorance of this etiological reason could lead to delayed diagnosis or even worsening hypertension due to the administration of steroids for idiopathic nerve palsy. Many authors recommend the measure of blood pressure in all patients with facial paralysis. It is essential to mention that we found several datasets that support PFNP as the first symptom of hypertension in children [28]. In our study, hypertension was detected in any patients. But measuring blood pressure is essential in every issue of PFNP.

PFNP, especially recurrent palsies, are uncommon disorder. In the literature, it was observed patients with recurrent PFNP, were diagnosed with celiac disease (CD) several months later. Because this observation and other neurological symptoms may be the only manifestation of atypical forms of CD. In our study, a 5-year-old girl suffered from CD for 12 months before developing PFNP for the first time; no other attacks were detected during follow-up [2, 4, 29]. Recurrent PFNP was seen in two cases, the first one was diagnosed as Melkersson–Rosenthal syndrome and the other one was considered idiopathic due to normal physical examination and laboratory and radiological test results except for B12 deficiency. Therefore this study cannot make causal inferences on the relation between B12 deficiency and PFNP. Nevertheless, there are many reported cases of neurological deficits due to B12 deficiency. One of these cases is a recurrent facial palsy in a 40-year-old woman who revealed primary Gougerot–Sjögren's syndrome. The onset of facial palsy has been linked with Gougerot–Sjögren's syndrome. The contribution of vitamin B12 deficiency is debatable in that case. So measuring B12 in cases of PFNP could be important, and thus supplying that patient with B12 could play a significant role in the treatment and prognosis [29].

Iatrogenic FNP cases are also reported. Complications can occur during mastoid surgery causing injury to the facial nerve. Early facial nerve exploration and neurolysis resulted in good facial nerve recovery. Some cases of PFNP secondary to superficial parotidectomy are also available in the literature [1, 2, 30]. In addition, bilateral facial nerve palsy secondary to the administration of high-dose paclitaxel was also reported in a woman with breast cancer [13]. In our study, there is one case of PFNP due to iatrogenic reasons (during cochlear implant surgery). At the same time, our study revealed a case of PFNP after tacrolimus treatment in a patient with renal transplantation**.**

MRI is beneficial in identifying brainstem pathology. High-resolution computed tomography scanning is better for evaluating the infratemporal portion of the nerve, especially in traumatic cases. Contrast-enhanced magnetic resonance imaging can identify sections of affected nerve in idiopathic facial palsy, but this test is not indicated in most children. More recent techniques such as constructive interference in steady-state and 3D-magnetization-prepared rapid gradient echo can also be used to evaluate anatomical details of the inner ear and facial nerve. It should be indicated especially in patients presenting with atypical clinical findings and not improving as expected [6, 21].

Electrophysiological analyses of the facial nerve and the mimic muscles can assist in diagnosis, assess the lesion severity, and aid in decision-making. An acute facial palsy is a valuable tool for predicting recovery. The American Academy of Otolaryngology-Head and Neck Surgery Foundation guideline recommends electrodiagnostic testing only for cases of complete paralysis. In contrast, the German and the Spanish guidelines recommend electrodiagnostic for all patients with Bell’s palsy. Electrophysiological analyses were done in only 12 patients. This could be due to the lack of cooperation in children, Electrophysiological analyses may detect the presence of voluntary motor unit action potentials. In traumatic cases, this proves that the facial nerve has not been completely transected. This finding directly impacts the decision to explore the lesion site [22].

The treatment of idiopathic PFNP is controversial. The use of steroids early at the onset of palsy (within 72 h) improves the prognosis and chances of complete recovery. This theory has been conducted depending on studies in adults. In children, the clinical benefit of steroids has not been proven yet. This is possible because most children with PFNP with or without the use of steroids resulted in complete recovery. Many studies demonstrate that children generally have better outcomes. Some research revealed a beneficial effect of using a steroid. In a recent randomized, double-blind, and placebo-controlled research on 496 patients with Bell’s palsy after 3 months, 83% of the patients in the corticosteroid group recovered compared to 64% in the placebo group. The same study showed after 9 months of follow-up this proportion increased to 94% for the corticosteroid group and 82% for the placebo group. The researchers thought depending on the result of this research that the treatment with steroids within 3 days after onset profoundly improves the chance for complete recovery at 3 or 9 months. In another study on 147 patients with peripheral facial palsy 44% received corticosteroids, which did not significantly improve the outcome [33]. In our study, steroid was started in 39 out of 57 patients, this could be due to a strong belief in the strong effects of steroids, and 46 of the children achieved full recovery under oral steroids or other required drugs according to the etiology within 1–7 months post-treatment.

Patients with Ramsay Hunt syndrome should be aggressively treated with intravenous administration of acyclovir plus steroids. Two recent reviews with acyclovir and steroids versus steroids alone, acyclovir versus steroids, and valacyclovir with steroids versus steroids. The study concluded that the results of all three trials were inconclusive about a short or long-term benefit and that a large, multicenter, randomized, controlled, and blinded study with a minimum follow-up of 1 year is required before a definite recommendation, however, acyclovir appears to be effective in patients with PFNP due to herpes zoster or VZV infection [34]. In our study, CMV, EBV, varicella-zoster, mycoplasma pneumonia, otitis media, otitis externa, and abscess led to PFNP in 11(19.2%). Patients with PFNP caused by infections recovered within 0.5–3 months after treatment.

In patients unable to close their eyes, appropriate eye care is needed to help avoid corneal abrasions. This care can be provided by using artificial tears, sun protection, and rarely tarsorrhaphy. In our study, all patients received artificial tears and eye care. Additional measures such as acupuncture and moxibustion could be applied. Though only limited experience has been reported with acupuncture for Bell’s palsy [9], several studies provide increasing evidence for the beneficial effect of acupuncture and moxibustion as an adjunctive treatment of Bell’s palsy [30–32]. In our study, none of the patients received acupuncture and moxibustion.

There are only a few controlled trials present on the real effectiveness of physical therapy for facial palsies. In a randomized trial on 50 patients with Bell’s palsy and a mean HBS of IV, mime therapy, including auto massage, relaxation exercises, inhibition of synkinesis, coordination exercises, or emotional expression exercises, resulted in improvement of facial stiffness [35–38]. In our study, physical rehabilitation programs were provided to all patients.

Not all cases of PFNP have resulted in complete recovery. There are cases with poor outcomes, so in these patients, further methods of treatment are applied as pulsatile electrical current (transcutaneous electrical stimulation) particularly, in chronic facial nerve damage long-term electrical stimulation may be beneficial. In a study on 12 patients with chronic PFNP and 5 cases due to iatrogenic causes, such stimulation was applied. There was an improvement in these patients. The beneficial effect was due to the facilitation of re-innervation through electrical stimulation. Another further way of treatment in poor prognosis patients is transmastoid decompression. In a study on 58 patients with PFNP having denervation exceeding 95%, transmastoid decompression of the facial nerve resulted in significant improvement of the HBS. Gold weight Implantation into the upper eyelids is also applied in poor prognosis patients a study regarding gold implantation on 16 patients with lagophthalmos due to PFNP resulted in a significant reduction of lagophthalmos and improved corneal coverage of 100%. Facial nerve cable grafting is also another treatment method that is applied in poor prognosis. In a retrospective study of 27 patients undergoing facial nerve grafting who had the nerve grafted to a site distal to the mental foramen had a better outcome than those with anastomosis proximal to the meatal foramen. The subperiosteal facial suspension (face lifting) In an observational study on five patients with an HBS of III–V face lifting resulted in a marked improvement in four of them [39–44]. These further method generally were applied on adult patients, in our study non of these methods were approached.

Assessing the prognosis of facial nerve paralysis can be difficult, especially in children, even if the possibility of a complete functional recovery is greater in pediatric cases than in adult ones. Facial nerve palsy can improve up to 1 year later. Complete palsy, Absent recovery by 3 weeks, Age > 60 years, Severe pain Ramsey Hunt syndrome, Presence of conditions causing secondary facial nerve palsy, and Reduction of the compound muscle action potential > 50% are considered to be Indicators for poor prognosis. The grade is very important when determining the prognosis. Patients with partial paralysis have a better prognosis. The II-degree, according to the House–Brackmann scale, has a good outcome, while the III and IV degrees are associated with moderate residual dysfunctions. The V and the VI degrees, instead, have a poor possibility of recovery. It has been reported that in about 5% of cases, the affected side may develop residual sequelae like contractures, spasms, and synkinesis. The latter, in particular, affects symmetry and facial expressiveness. The most common synkinesis affects the eye and mouth muscles: during a voluntary movement of the mouth, for example, a smile, there could be an involuntary eye closure and vice versa. A similar phenomenon can occur with the autonomic fibers: for example, when eating, the activation of salivation also causes lacrimation (a phenomenon known as “crocodile tears”). No patient revealed such residual sequelae despite unrecovered 4 cases and partial recovery in 2 patients in our studies. In our study, the prognosis of PFNP is good with complete recovery in about 80.7% of the cases, 3.5% experienced some kind of improvement, and 7% remained with severe sequelae, our results are similar to Joseph et al. results that showed complete recovery in about 80% of the cases, 15% experienced some improvement 5% were with severe sequelae [34, 45, 46].

In conclusion, peripheral facial nerve palsy is a rare condition in children with different causes. It could be idiopathic, congenital, or due to infectious, traumatic, neoplastic, and immune reasons. So, when a child presents with facial palsy, a complete clinical history and a detailed clinical examination are recommended. Giving attention to the red flag is very important. Peripheral facial nerve palsy in children is considered to have a good prognosis.

## Limitations of the study

As this study did not have a control group without steroid treatment, a comparative assessment of steroid treatment was unavailable.

## Data Availability

At Ass Prof. GGM repository. The datasets used and/or analyzed during the current study are available from the corresponding author upon reasonable request.

## Abbreviation

PFNP: Peripheral facial nerve palsy
